# Unlocking Parkinson’s disease: harnessing the untapped potential of fatty acid metabolism in disease pathogenesis

**DOI:** 10.3389/fimmu.2026.1937115

**Published:** 2026-09-15

**Authors:** Rong Hu, Yaru Song, Ting Liu, Xinyu He, Youming Qiu, Fanjing Zhong, Xiao Huang, Junyun Huang, Zhigang Jiao

**Affiliations:** 1 Department of Laboratory Medicine, First Affiliated Hospital of Gannan Medical University, Ganzhou, China; 2 College of Medical Technology, Gannan Medical University, Ganzhou, China; 3 The First School of Clinical Medicine, Gannan Medical University, Ganzhou, China; 4 Ganzhou Central Blood Station, Ganzhou, China; 5 Gannan Branch of National Geriatric Center, First Affiliated Hospital of Gannan Medical University, Ganzhou, China

**Keywords:** alpha-synuclein, fatty acids, neuroinflammation, oxidative stress, Parkinson’s disease

## Abstract

Parkinson’s disease (PD) is the second most common neurodegenerative disorder, and its rising incidence poses a growing burden on productivity and healthcare systems, particularly in the context of global population aging. Despite substantial advances in research, the pathogenesis of PD remains incompletely understood, and no curative or effective preventive therapies are currently available. Emerging evidence suggests a potential link between fatty acid (FA) metabolism and PD pathogenesis, positioning the modulation of FA metabolic pathways as a promising therapeutic strategy. This review examines the interplay between FA metabolism and key pathological processes, including oxidative stress, neuroinflammation, and the abnormal aggregation of alpha-synuclein, within the context of PD. By integrating these perspectives, the article aims to advance understanding of disease mechanisms and identify novel opportunities for prevention, early diagnosis, and targeted treatment.

## Highlights

- Fatty acids connect key PD processes, offering a new insight to understand the etiology of PD.
- Understanding fatty acid metabolism may reveal new mechanisms for PD.
- Modulating fatty acid pathways could enable novel prevention and treatment strategies for PD.

## 1 Introduction

Parkinson’s disease (PD) is the second most prevalent neurodegenerative disorder worldwide, a leading cause of disability, and among the most rapidly increasing neurological diseases (1). Projections estimate that its prevalence will double in the next generation, affecting approximately 9.6 million individuals (2). This escalating trend is expected to place a substantial socioeconomic burden on the global community. Consequently, the development of effective strategies for the prevention, early diagnosis, and treatment of PD is of critical importance (3). At present, no available therapy can cure or halt the progression of the disease, and existing interventions primarily aim to relieve symptoms. Furthermore, PD diagnosis largely depends on the identification of motor symptoms, with no widely available diagnostic tools that are both highly sensitive and specific. These limitations underscore an urgent need for innovative, more effective approaches to improve prevention, enhance diagnostic accuracy, and develop disease-modifying treatments.

Two hallmark pathological features of PD are the progressive degeneration of dopaminergic neurons in the substantia nigra pars compacta and the abnormal accumulation of α-synuclein (α-syn). Clinically, PD is characterized by cardinal motor symptoms such as resting tremor, bradykinesia, rigidity, and postural instability, alongside a wide spectrum of non-motor symptoms, including autonomic dysfunction, sleep disturbances, and cognitive decline. These manifestations can substantially impair patients’ quality of life to varying degrees (4). Epidemiological evidence indicates that PD is a multifactorial disorder arising from complex interactions among environmental, genetic, and lifestyle factors, which converge on key pathogenic mechanisms, including mitochondrial dysfunction, oxidative stress, protein aggregation, impaired autophagy, and neuroinflammation (5). Age is the most significant risk factor, with the average onset occurring at approximately 60 years, and the likelihood of developing PD increasing steadily with advancing age (6). Environmental and lifestyle influences also play a critical role. Exposure to neurotoxic agents such as 1-methyl-4-phenyl-1,2,3,6-tetrahydropyridine (MPTP), paraquat, insecticides, and rotenone has been linked to a heightened risk of PD (7–9). Additional risk factors include dairy consumption, head trauma, a history of melanoma, and chronic exposure to heavy metals (10, 11). The loss of function of the Melanocortin 1 receptor (MC1R) has been linked to an increased risk of melanoma and is observed to have diminished activity in PD models and patients. Mechanistic studies have demonstrated that the loss of MC1R function impedes the nuclear translocation of nuclear factor erythroid 2-related factor 2 (Nrf2), thereby disrupting the redox system and enhancing fatty acid oxidation in PD (12). In contrast, cigarette smoking, caffeine consumption, elevated serum urate levels, regular physical activity, and the use of ibuprofen and certain other commonly prescribed medications have been associated with a reduced risk (13–15). Eicosanoyl-5-hydroxytryptamide, a fatty acid derivative of serotonin present in coffee, inhibits protein phosphatase 2A (PP2A) methylesterase, thereby maintaining PP2A in a highly active methylated state and facilitating the dephosphorylation of α-syn (16). The deposition of phosphorylated α-syn is a main pathological feature of PD patients and hyper-phosphorylated α-syn tends to fibrillize more than unmodified α-syn, which was neurotoxin compare than the unmodified α-syn (17, 18). Therefore, the inhibition of hyper-phosphorylated α-syn may be beneficial for PD patients.

Recent studies have increasingly highlighted dysregulated lipid profiles in patients with PD, with particular attention to the relationship between fatty acids (FAs) and PD risk (19). Liquid chromatography–mass spectrometry analyses have demonstrated significant alterations in the carnitine shuttle within the sebum of PD patients compared to healthy controls. These modifications seem to be more closely linked to the progression of the disease than to the effects of pharmacological treatment for PD (20). Given the carnitine shuttle’s key role in energy metabolism—facilitating the β-oxidation of long-chain fatty acids (LCFA) by transporting them into the mitochondria via acyl-carnitine substrates (21)—disruptions in FA biosynthesis and metabolic pathways may plausibly contribute to PD pathogenesis. Altered FA composition has also been reported in lipid rafts of the frontal cortex in PD patients, resulting in a more ordered, viscous fluid state. Such changes are associated with impaired neuronal function, synaptic transmission, and neurotransmitter signaling (22). Similarly, lipidomic profiling of the midbrain in a manganese-induced mouse model of PD demonstrated marked changes in the composition of LCFAs (12 < C ≤ 21) and ultra-long-chain FAs (23). Moreover, several PD-associated genetic mutations, including those in Parkin and LRRK2, have been implicated in FA metabolic regulation (24, 25). Dietary studies further suggest that both the quantity and type of FA intake, as well as the activity of FA-regulatory proteins, such as monoacylglycerol lipase, stearoyl coenzyme A desaturase-2, FA-binding proteins (FABPs), and FA translocator (FAT/CD36), are linked to PD risk and progression (26–31). Collectively, these findings indicate that both intracerebral FA metabolism and exogenous FA intake are closely associated with PD development, and their modulation may offer novel, non-neurocentric therapeutic strategies for disease intervention.

## 2 Neuronal FA synthesis and degradation

Neurons consist of the soma, axon, and dendrites, the latter featuring numerous branches and spines. This complex morphology demands a substantial supply of membrane lipids to maintain cellular integrity and function. FAs, as essential lipid components, are critical for sustaining membrane composition. This outcome suggests that neuron contain more FAs or have a higher turnover rate of FAs. Fatty acids are classified by chain length and saturation (**Figure 1**). Neurons acquire FAs from external sources, via *de novo* synthesis, and through the breakdown of intracellular triacylglycerol (TAGs) and phospholipids. Saturated FAs (SFAs) are indispensable for brain development and, in adults, serve as structural building blocks for nearly all lipid classes, as precursors of bioactive lipids, and as substrates for fatty acylation of proteins. However, excessive levels of long-chain SFAs or very long-chain SFAs (VLCFAs) can be neurotoxic. For example, palmitic acid (PA), a long-chain SFA, has been shown to induce anxiety-like behaviors in mice (32, 33). Elevated levels of VLCFAs have been observed in patients with X-linked adrenoleukodystrophy (X-ALD) or aceruloplasminemia, although the mechanisms by which VLCFAs induce neurodegeneration remain incompletely understood (34, 35). Conversely, long-chain ω-3 and ω-6 FAs exert protective effects in the central nervous system (CNS) (36). For further insights into the role and mechanisms of SCFAs in PD, readers are referred to a recent review (37). Notably, neurons have a limited capacity to synthesize polyunsaturated FAs (PUFAs) such as 20:4 ω-6 and 22:6 ω-3 from their precursors (18:2 ω-6, 20:4 ω-6, 18:3 ω-3, and 20:5 ω-3) compared with SFAs. Both cerebral and cerebellar neurons exhibit minimal desaturation of long-chain ω-3 or ω-6 FAs due to low Δ-4 desaturase activity relative to astrocytes. Instead, neurons readily acquire long-chain ω-3 and ω-6 FAs from astrocytes, which possess higher elongase and desaturase activity and have greater access to precursor FAs from the circulation owing to their strategic anatomical position (38).

**Figure 1:**
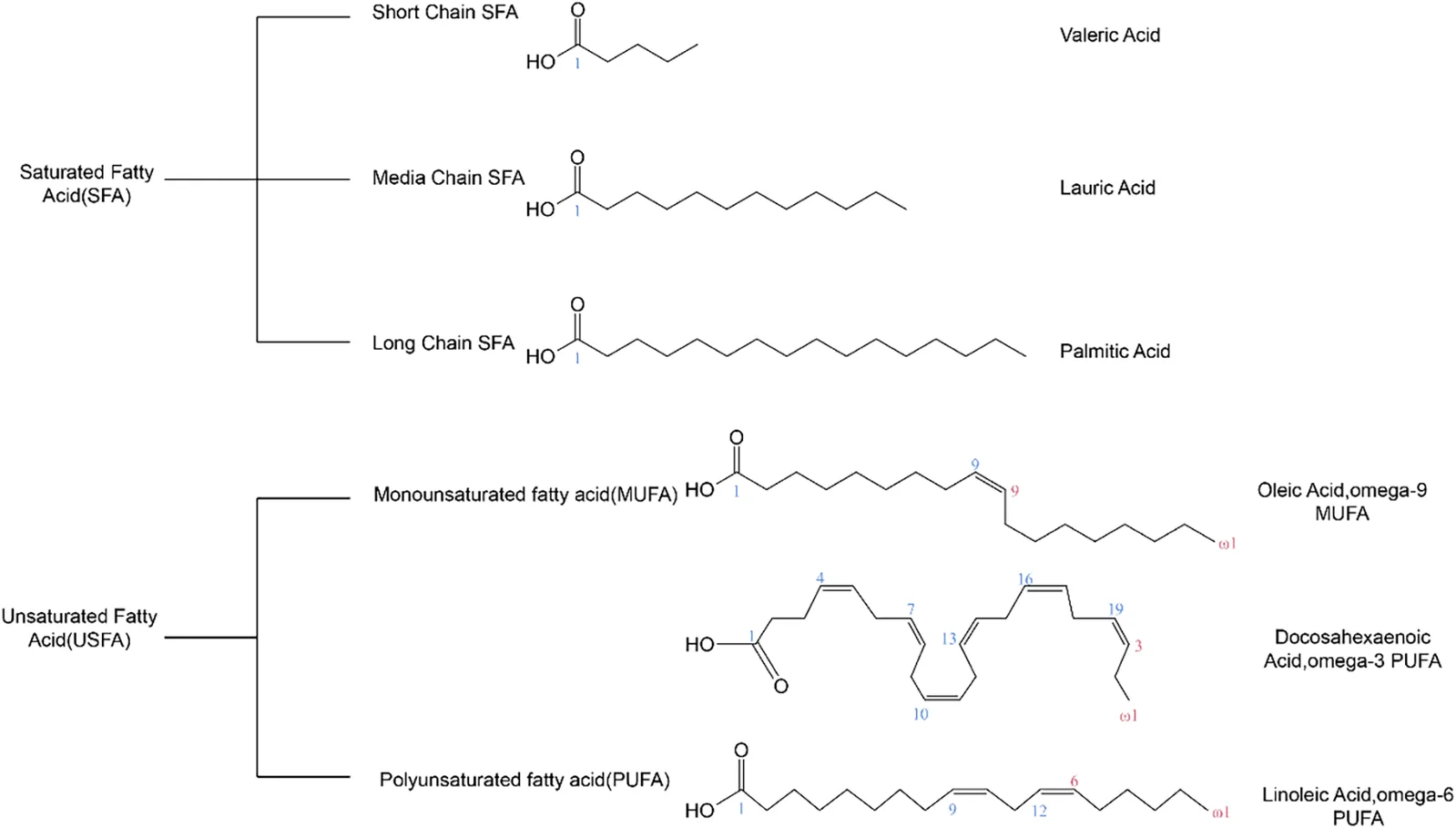
Classification and representative structures of fatty acids. Fatty acids are categorized as saturated fatty acids (SFAs) or unsaturated fatty acids (USFAs). SFAs, which lack double bonds, are grouped by chain length (short-, medium-, long-chain; exemplified by valeric, lauric, and palmitic acid, respectively). USFAs contain one or more double bonds and include monounsaturated (MUFAs; oleic acid, ω-9) and polyunsaturated [PUFAs; docosahexaenoic acid (DHA, ω-3) and linoleic acid (LA, ω-6)] forms. Representative chemical structures for each subclass are shown. Created with Adobe Illustrator 2020.

*De novo* FA synthesis is also an important anabolic pathway in neurons. This cytosolic process begins with the carboxylation of acetyl-CoA to malonyl-CoA by acetyl-CoA carboxylase (ACC), a rate-limiting enzyme in lipogenesis with two isoforms: ACC1 and ACC2. ACC1, located in the cytoplasm, is primarily involved in adipogenesis, whereas ACC2, located on the outer mitochondrial membrane, regulates FA β-oxidation. Given that neuronal β-oxidation capacity is limited (39), it is likely that ACC1 predominates in neurons. ACC1 promotes FA synthesis and concurrently inhibits FA oxidation (40). However, this pathway may be metabolically suboptimal for neurons, as it consumes ATP. Neuronal activity is sustained primarily by ATP derived from circulating glucose via glycolysis and oxidative phosphorylation (41, 42). Additionally, neurons can obtain FAs through the degradation of damaged intracellular lipids via autophagy or lipases such as cytosolic phospholipase A2 (39, 43). Autophagy flux in neurons is activated under oxidative stress, leading to the degradation of damaged membrane lipids and the release of free FAs (FFAs), which are subsequently re-esterified into new lipids and incorporated into cellular membranes (44).

In addition to supporting essential neuronal functions, neurons have a limited capacity for lipid storage and for generating energy from FAs. Excess FFAs can exert neurotoxic effects by impairing mitochondrial function or disrupting membrane integrity through their lytic activity (39, 45, 46). As a key adaptive mechanism against lipotoxicity, surplus FFAs are either sequestered within intracellular lipid droplets (LDs) for storage or integrated into apolipoprotein E (ApoE)-containing lipid particles; these LDs or particles are then shuttled to astrocytes for further metabolic processing or clearance (39, 44). Astrocytes are metabolically coupled to neurons, and this interaction plays a crucial role in mitigating neuronal vulnerability arising from limited energy reserves in the brain (47) (**Figure 2**). Overactive neurons produce excessive amounts of toxic FAs that are exported via ATP-binding cassette transporter protein 1 or ApoE-positive lipid particles. Astrocytes take up these FAs through endocytosis, storing them in LDs (48–50). Within astrocytes, FAs stored in LDs can be mobilized for β-oxidation, providing an alternative energy source for neurons, while simultaneously upregulating metabolic and detoxification genes in response to oxidative stress (39). However, LDs are central to intercellular metabolic coupling and attenuate lipid peroxidation–induced toxicity in neurons by converting FFAs into TAGs and sequestering them in a storage form (51). Therefore, the regulation of lipid droplet formation in astrocytes may affect brain function. For example, in a PD model, LA demonstrated antioxidant properties by promoting LD biogenesis and enhancing autophagy/lipophagy flux via the diacylglycerolacyltransferase-2 (DGAT-2) enzyme (52). Conversely, in primary mouse astrocytes with FABP7 knockdown, an essential protein for LD formation, ROS-induced LD accumulation was markedly reduced, resulting in greater reactive oxygen species (ROS) toxicity and increased apoptosis (53).

**Figure 2:**
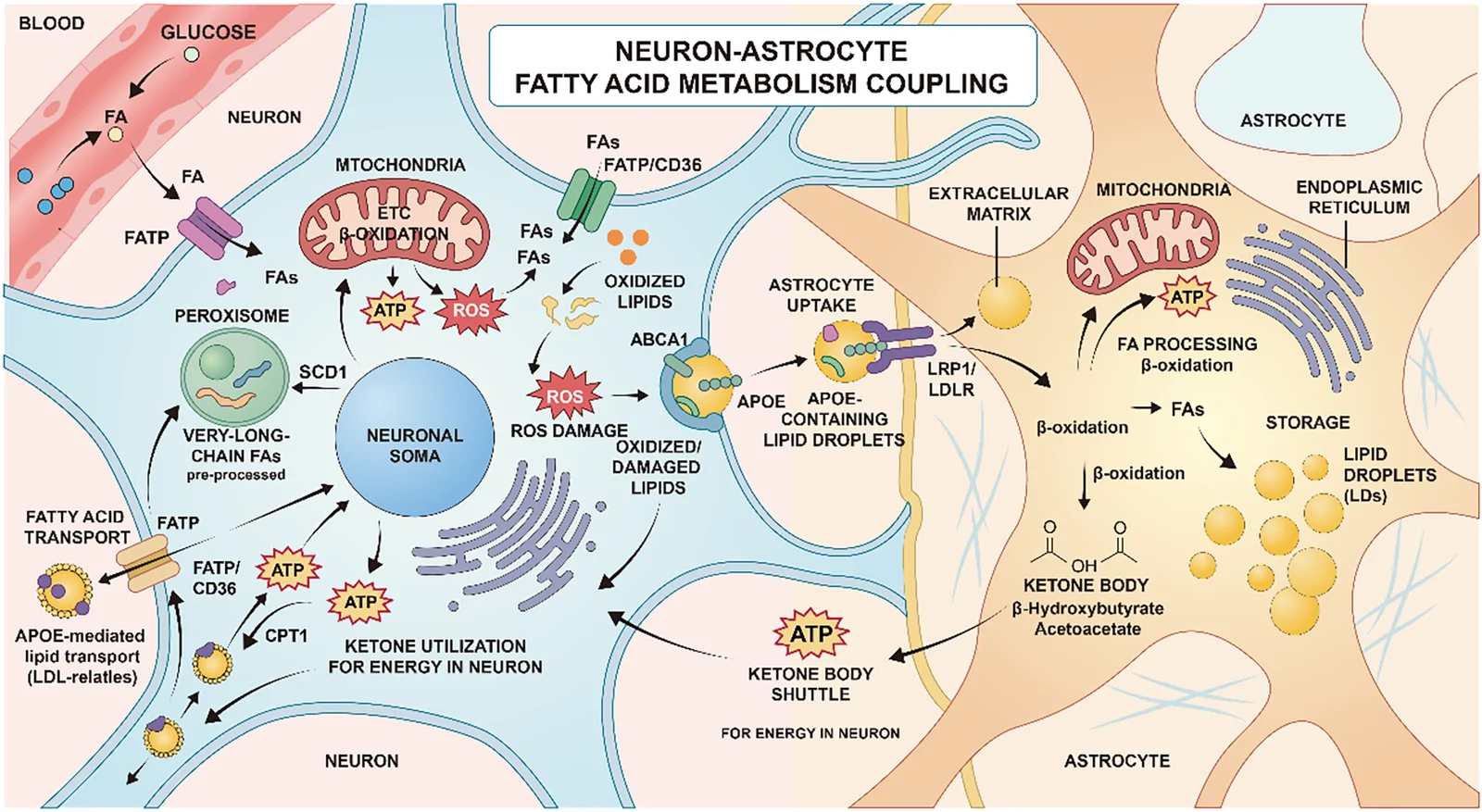
Neuron–astrocyte coupling in fatty acid metabolism. Neurons assimilate circulating FAs through the FATP/CD36 pathway, facilitating their mitochondrial β-oxidation to produce ATP, while concurrently generating ROS. Surplus or oxidized FAs are transported to astrocytes via lipid particles containing ABCA1-APOE. Astrocytes internalize these lipid particles through LRP1/LDLR receptors, subsequently storing them in LDs. Within astrocytes, FAs are processed via β-oxidation to generate ATP or are converted into ketone bodies (β-hydroxybutyrate, acetoacetate) to supply energy to neurons. Additionally, the peroxisomal metabolism of very-long-chain FAs and the desaturation process mediated by SCD1 in neurons are illustrated, emphasizing the role of this metabolic coupling in mitigating lipotoxicity and enhancing neuronal bioenergetics. Created with Adobe Illustrator 2020.

However, if brain FA levels exceed the metabolic coupling capacity of astrocytes, or if astrocytic FA processing is impaired, or if neuronal FFA transfer is blocked, lipid homeostasis in the brain becomes disrupted, triggering pathological processes such as oxidative stress and neuroinflammation (54). Studies have shown that astrocytes exposed to FA overload exhibit impaired Ca^2+^ signaling, which increases neuronal susceptibility to inflammatory injury and disrupts neurotransmitter release and synaptic function (55). In a mouse model of astrocyte-specific oxidative phosphorylation deficiency, abnormal LD accumulation was observed. An imbalance between FA and acetyl–coenzyme A levels activated the acetylation of signal transducer and activator of transcription 3, thereby inducing reactive astrocytogenesis (56). Moreover, neurons co-cultured with C9 mutant astrocytes, characterized by reduced antioxidant capacity, showed a higher proportion of ROS positivity compared to controls (57). Recent findings also indicate that astrocyte–neuron interactions are regionally specific, with significant differences in astrocytic modulation of neurons in the ventral midbrain versus the striatum (58). This suggests that the FA metabolic coupling between astrocytes and neurons may likewise display regional heterogeneity. However, research on region-dependent astrocyte–neuron functions remains limited. Investigating the mechanisms of FA metabolism coupling in this context, and enhancing the astrocytic capacity to mitigate neuronal oxidative stress or lipotoxicity, may represent promising therapeutic strategies for PD. In the future, refining our understanding of regional differences in astrocyte–neuron FA metabolic coupling could facilitate the development of more precise interventions for PD.

## 3 Functions of neuronal FAs

As fundamental structural components of the body, FAs play critical roles in energy metabolism and cellular signaling pathways. Within mitochondria, FAs undergo β-oxidation to generate acetyl-CoA, which enters the tricarboxylic acid cycle to support ATP production; alternatively, FAs serve as structural backbones for the synthesis of TAG, phospholipids, or cholesterol esters (59). Lipids are major constituents of brain tissue, accounting for approximately 60% of its dry weight (60). Brain lipids are closely involved in structural development, neurogenesis, synaptogenesis, and myelin formation, and are essential for maintaining brain integrity (61–63). FAs form the structural basis of neuronal membranes and myelin sheaths, preserving membrane fluidity and facilitating neurotransmitter transmission; they also act as energy reservoirs to maintain energy homeostasis in the nervous system (64). Furthermore, brain FAs function as signaling molecules that modulate diverse neural processes by regulating intracellular signaling pathways. For example, FFAs in neurons reduce neuronal excitability in the occipital cortex by regulating Na^+^/K^+^-ATPase activity in response to cerebral norepinephrine exposure (65). In another study, sodium butyrate improved cognitive impairment in diabetic mice by restoring PRKN/Parkin-mediated mitophagy in the hippocampus, achieved through inhibition of RELA nuclear translocation and direct suppression of HDAC8 activity in the nucleus (66).

The CNS contains a diverse array of FAs, broadly classified into SFAs, monounsaturated FAs, and PUFAs (67). Saturated and monounsaturated FAs can be synthesized *de novo* within the brain (68). PUFAs, by contrast, are essential FAs that must be acquired from the circulation and transported across the blood–brain barrier, primarily via specialized transporter proteins and/or passive diffusion through endothelial membranes (69–71). PUFAs are further subdivided into two major families based on the position of the first double bond from the methyl end of the carbon chain: ω-3 and ω-6 PUFAs (72).

### 3.1 ω-3 PUFAs

The principal ω-3 PUFAs include alpha-linolenic acid, eicosapentaenoic acid, docosapentaenoic acid, and docosahexaenoic acid (DHA). Numerous studies have demonstrated that ω-3 PUFAs exert anti-inflammatory, antioxidant, pro-apoptotic, and lipid-metabolism–regulating effects (73–75). DHA, the most abundant ω-3 PUFA in the brain, constitutes approximately 12%–15% of total brain FA content and is critical for brain growth and functional maturation (76). Predominantly localized in neuronal membranes within the gray matter, DHA has a concentration that correlates positively with cognitive performance (77). Experimental evidence suggests that docosahexaenoic acid (DHA) enhances tyrosine hydroxylase activity by promoting its phosphorylation through protein kinase A (PKA)- and protein kinase C (PKC)-dependent pathways. This mechanism helps to preserve dopamine levels and maintain motor function in the medial forebrain bundle in a 6-hydroxydopamine (6-OHDA) model of advanced-stage PD in rats (78). In this study, DHA was administered at a dosage of 30 mg/kg body weight via intraperitoneal injection for 15 consecutive days, with the initial injection occurring prior to the 6-OHDA infusion (79, 80). It is important to note that other studies have reported that elevated levels of DHA may enhance the neurotoxicity of α-syn and facilitate neurodegeneration. Yakunin et al. found that both A53T transgenic mice on a DHA-enriched diet (~0.69% w/w for 95 or 230 days) and cultured dopaminergic neurons exposed to physiological concentrations of DHA exhibited upregulated expression of α-syn and increased α-syn accumulation in the cortical region. DHA’s contradictory neuroprotective or neurotoxic effects are explained by context-dependent mechanisms, DHA dose, experimental model, or timing of intervention (81, 82). Its biological outcome is tightly regulated by concentration, exposure duration, *in vitro/in vivo* models, and antioxidant presence (83) (**Table 1**). Detailed information about these roles is provided in each section of this review.

**Table 1:** Summary of findings related to the effect of DHA in neurodegeneration models.

Cell/Tissue model	Disease model	DHA administration	Treatment	Primary outcomes	Ref
PC12 cell line	General neuronal injury model	DHA-phosphatidylserine 10 and 40 µg/mL for 24 h	200 µmol/L H_2_O_2_ or 150 µmol/L t-BHP for 4 h	↑SOD activity and Total antioxidative capacity; ↓Caspase-3 and Caspase-9 genes;↑ Bcl-2 protein;↓Bax protein	(84)
SH-SY5Y cell line	PD model	100 μmol incubation with 6-OHDA for 24 h	50 μmol 6-OHDA for 24 h	A less protective effect on SH-SY5Y induced by 6-OHDA, such as cellular viability, apoptosis, the mitochondrial morphology and the expression of BDNF and GDNF gene and protein	(85)
N27 cell line	PD model	25 μmol incubation with 6-OHDA for 6 h	150 μmol 6-OHDA for 6 h	↑ cellular viability; ↓the level of ROS, lipid peroxidation, NO production, TNF-α, IL-1β, IL-6, PINK1 and Parkin	(86)
N27 cell line	General neuronal injury model	50 μmol incubation with soluble α-Syn at 37 °C for 70 h	50 μmol αSyn accumulation or the mixture of DHA and αSyn	↑Aggregated and insoluble α-Syn; ↓spend time of aggregation α-Syn	(87)
SD rats (age from 2 to 3 months, male)	PD model	intraperitoneal 300 mg/kg weight for two weeks	Stereotaxic infusion of 6-OHDA (30 μg)	Improved the movement function; ↑TH and Ser^40^-phospho TH; ↑dopamine concentration; ↑activity of PKA and PKC	(78)
SD rats (age in 8 months, female)	Aged model	Combine with diet (high-fat diet, 45 kcal% fat; DHA, 1% w/w)	high-fat diet, 45 kcal% fat	↓the number of GFAP- and IBA1-positive cells in hippocampus; ↓TNF-α, iNOS and COX content; ↑ GPx activity and GSH content; ↑Nrf2 and HO-1 protein expression; ↓3-NT, APP, BACE1, p-tau (Ser396) protein expression; ↓phosphorylation levels of JNK and IKK; ↑phosphorylation levels of Akt and GSK3β; improved the synaptic function	(88)
SD rats (170 g–220 g, male)	PD model	intragastric daily,50 mg/kg for 20 weeks	stereotaxic infusion of 6-OHDA in striatum (7.5 μg)	↓ motor impairments; ↑ the number of TH-positive cells and ↓ the number of GFAP- and IBA1-positive cells in striatum and SNPc	(89)
Wistar rats (70 days, male)	PD model	intragastric daily for 50 days; DHA: 0.36g/kg weight	stereotaxic infusion of 6-OHDA in striatum (10 μg)	↑the number of TH-positive cells in striatum and SNPc; ↓the number of OX42- positive cells in striatum and SNPc;↓the number of GFAP-and iNOS-positive cells in striatum and SNcv;↓the number of GFAP- and iNOS-positive cells in SNcd;	(90)
Thy1-αSyn mice	PD model	Combine with diet (DHA, ~0.8 g/kg for 10 months)		↑DHA content and insoluble human αsyn in frontal cortex; improved the survival of Thy1-αSyn mice; ↑SNAP25; TH, drebrin, NeuN and septin protein expression and motor impairments	(91)
Thy1-αSyn mice	PD model	Combine with diet (DHA, ~0.78g/kg for 10 months)		↑ the DHA level in the plasma; the TH-positive cell and varicosities in myenteric plexus of Thy1-αSyn mice; no effect on the accumulation of αSyn; ↑GLP-1, Nrf2	(92)
C57BL/6 mice (age from 7 to 8 weeks, male)	Depression model	Oral with drinking, 100 mg/kg weight for 45 days	2% dextran sulfate sodium (DSS)	↑weight; improved the movement function impaired by 2% DSS; ↑ZO-1, 5-HT, PSD95, and snaptophysin protein; ↓NLRP3, ASC protein;	(93)
C57BL/6 mice (age from 7 to 8 weeks, male)	PD model	exercise combine with/or diet at 4 weeks post-lesion (DHA, 0.725% w/w) for 28 days	stereotaxic infusion of 6-OHDA (4 μg)	↓motor impairments; ↑dopamine concentration; the number of TH-positive cells in striatum rather than SNPc	(94)
APP/PS1 mice (3 months old) and SAMP8 mice (4 months old)	General neuronal injury model	Combine with diet (DHA, ~0.233g/kg for ten months)		↓TNF-α, IL-2, IL-6 and MCP-1, the relative abundance of Proteobacteria in gut; improved the learning and memory function	(95)
A53T a-Syn tg mouse and MN9D	PD model	Combine with diet (DHA, ~0.69% w/w) for 95 ± 5 or 230 days) and BSA-DHA (at 50 μmol and 200 μmol) for 16 h		↑a-Syn accumulation in the brain stem and the cortex at 230 days; ↑the activation of retinoic X receptor (RXR) and peroxisome proliferator-activated receptor g2 (PPARg2) in the MN9D with a-Syn over expressing	(80)
Elovl2^−/−^ mice (20–25 weeks)	General neuronal injury model	Combine with diet (DHA, ~1% w/w) for 3 months		↑Arc-1 gene expression and protein; ↓IL-1β and Casp1 mRNA and protein, Iba1^+^ and TMEM-119^+^ cells in the cerebral cortex	(96)

Thy1-αSyn mice, overexpresses human α-synuclein under the Thy1 promoter; APP/PS1 mice, double transgenic mice expressing a chimeric mouse/human amyloid precursor protein (Mo/HuAPP695swe) and a mutant human presenilin 1 (PS1-dE9); SAMP8 mice, a naturally occurring mouse line that displays a phenotype of accelerated aging; A53T a-Syn mice, expressed the mutant human A53T alpha-synuclein under the direction of the mouse prion protein promoter; Elovl2^−/−^ mice, the key enzyme deficient for DHA synthesis; PC12, derived from a transplantable rat pheochromocytoma; SH-SY5Y, a thrice cloned subline of the neuroblastoma cell line SK-N-SH; N27, Rat Dopaminergic Neural Cell Line; MN9D, Mice Dopaminergic Neural Cell Line; t-BHP, tert-Butyl Hydroperoxide. "↑" indicates significantly increased; "↓" indicates significantly decreased.

### 3.2 ω-6 PUFAs

The main ω-6 PUFAs are linoleic acid (18:2; ω-6; LA) and arachidonic acid (20:4; ω-6; AA). AA exhibits neurotoxic potential and serves as a precursor for multiple bioactive lipid mediators, including prostaglandins, leukotrienes, thromboxanes, and hydroxyeicosatetraenoic acids, which are involved in pro-inflammatory processes (97–99).

The role of FAs in neurodegenerative diseases has gained increasing attention with advances in lipidomics and proteomics. In the central nervous system, FA metabolism is closely linked to several pathogenic mechanisms of PD, influencing disease onset and progression through multiple pathways. For example, FA oxidation products have been shown to promote abnormal protein deposition, thereby accelerating the course of neurodegenerative disorders, including PD and Alzheimer’s disease (100). Moreover, the dietary ratio of ω-6 to ω-3 PUFAs has a direct impact on neuroinflammatory processes, with a higher proportion of ω-6 PUFAs promoting the expression of pro-inflammatory mediators in the brain (101). Experimental evidence also suggests that FAs extracted from sea cucumbers can attenuate 6-OHDA-induced oxidative stress and α-syn aggregation in *Caenorhabditis elegans* PD models at appropriate doses, highlighting their potential as a nutritional therapeutic approach for PD (102). Collectively, these findings suggest that neuronal damage mediated by oxidative stress, protein aggregation, and neuroinflammation may be mitigated through targeted modulation of FA type, metabolism, and intake.

## 4 FAs and oxidative stress in PD

The brain accounts for a substantial proportion of the body’s total energy consumption, and this high metabolic demand is accompanied by the generation of large amounts of ROS (103). Neurons are particularly vulnerable due to their relatively weak antioxidant defense mechanisms (104). First, their phospholipid membrane turnover rate is markedly lower than that of other cell types, which limits their ability to adapt to oxidative stress (105). Second, neurons exhibit high levels of the system xc^−^ cystine/glutamate antiporter and low levels of γ-glutamate–cysteine ligase, resulting in reduced glutathione levels, approximately 50% lower than in other cell types, which further compromises their resistance to oxidative stress (106, 107).

Although lipid peroxidation is well established in the pathogenesis of PD (108), the precise molecular mechanisms and the specific roles of FAs remain to be elucidated. FA peroxidation products can exert both cytotoxic and cytoprotective effects, with the ultimate cellular outcome determined by the FA substrate (e.g., ω-3 versus ω-6 PUFAs) and the pathway involved (e.g., enzymatic versus non-enzymatic) (**Figure 3**). Lipid peroxidation occurs when oxidants such as free radicals interact with FAs containing carbon–carbon double bonds, particularly PUFAs (109). Due to their high sensitivity to ROS, PUFAs are especially prone to oxidative stress, producing a range of neurotoxic compounds (110). For example, the ω-6 PUFAs LA and AA are readily oxidized to 4-hydroxynonenal, a reactive aldehyde that disrupts the BBB and contributes to PD pathogenesis. Furthermore, markedly elevated levels of lipid peroxidation end-products in glutathione peroxidase 4 (GPX4)-deficient mice activate ferroptosis signaling and promote α-syn aggregation, leading to dopaminergic neuronal dysfunction (108). Peroxidation can also occur through enzymatic pathways involving lipoxygenases (LOXs), cyclooxygenases (COXs), and cytochrome P450 (CYP) enzymes (111). For instance, ferroptosis—an iron-dependent form of regulated cell death—is initiated by the accumulation of lipid peroxides (LPOs), whose formation is mediated by three key enzyme families. During ferroptosis progression, long-chain acyl-CoA synthetase 4 (ACSL4) preferentially activates PUFAs, most notably arachidonic acid (AA) and adrenic acid (AdA, 22:4n-6), for integration into phosphatidylethanolamines (PEs) and phosphatidylcholines (PCs). These PUFA-containing phospholipids (PUFA-PLs) are highly susceptible to iron-catalyzed peroxidation via the Fenton reaction. Critically, three enzyme classes—lipoxygenases (LOXs), cyclooxygenases (COXs), and cytochrome P450 enzymes (CYPs)—catalyze the peroxidation of PUFA-PLs. This enzymatic cascade generates LPOs that ultimately trigger ferroptosis (112, 113).

**Figure 3:**
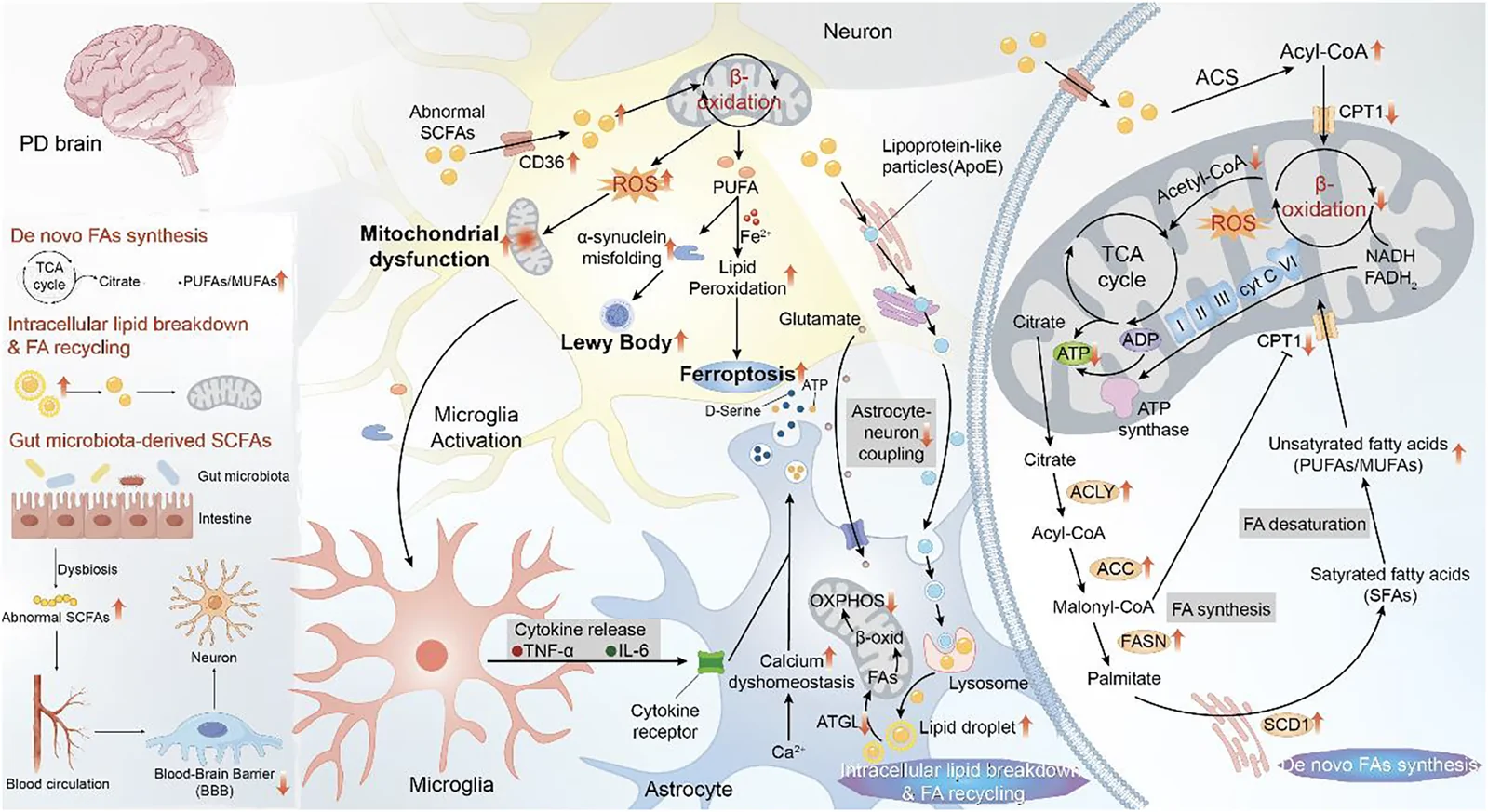
Dysregulation of fatty acid metabolism in PD pathogenesis. This schematic depicts how altered FA metabolism contributes to PD progression, spanning systemic and brain-intrinsic mechanisms. In the periphery, gut dysbiosis leads to abnormal short-chain FAs (SCFAs) and impaired blood–brain barrier (BBB) integrity, driving altered FA uptake into the brain. In neurons, increased CD36-mediated SCFA uptake and dysregulated β-oxidation promote mitochondrial dysfunction, reactive oxygen species (ROS) production, and lipid peroxidation, exacerbating α-synuclein misfolding, Lewy body formation, and ferroptosis. *De novo* FA synthesis is upregulated via ACLY, ACC, and FASN, increasing saturated (SFAs) and unsaturated (PUFAs/MUFAs) FA pools, while reduced CPT1 activity impairs mitochondrial FA import. In astrocytes, impaired lipid droplet turnover, calcium dyshomeostasis, and reduced β-oxidation disrupt the neuron-astrocyte coupling, while activated microglia release pro-inflammatory cytokines (TNF-α, IL-6) to amplify neurotoxicity. Collectively, these metabolic derangements drive glutamate/D-serine dysregulation, neuronal dysfunction, and PD pathogenesis. Created with Adobe Illustrator 2020.

Notably, mutations in CHCHD2, a recognized cause of familial PD, have been shown to preferentially impair the tricarboxylic acid cycle rate-limiting enzyme α-ketoglutarate dehydrogenase. This impairment disrupts the conversion of α-ketoglutarate to succinate, resulting in increased lipid peroxidation and phosphorylated α-syn accumulation (114). Consequently, antioxidants such as cannabidiol, wolfberry glycopeptide, and chlorogenic acid show potential neuroprotective effects in PD by enhancing antioxidant defenses, reducing FA peroxidation, and limiting lipid peroxidation build-up in the midbrain–striatal region (115–118).

In addition to exogenous antioxidants, astrocytes possess a stronger intrinsic antioxidant capacity than neurons. The Nrf2–Keap1–ARE signaling axis represents a key endogenous antioxidant system in astrocytes, conferring significant neuroprotective effects (119, 120). DJ-1, encoded by the PARK7 gene, functions as a critical oxidative stress regulator in astrocytes, modulating transcription factors such as Nrf2, PI3K/PKB, and p53 to counteract oxidative damage in the brain (121, 122). Astrocytes take up cystine via the cystine/glutamate exchange transporter (xCT) to synthesize glutathione, thereby maintaining redox homeostasis and slowing PD progression (123, 124). Moreover, astrocytes facilitate the uptake of dehydroascorbic acid, the oxidized form of ascorbate, from the extracellular environment and subsequently recycle it back to ascorbic acid. The regenerated ascorbic acid not only fulfills its intrinsic requirements but also serves as an antioxidant resource released into the extracellular space. It is subsequently taken up by neurons, where it provides protection against the toxicity induced by fatty acid oxidation (125). However, in the absence of protective reducing agents due to dysfunction of the Nrf2–Keap1–ARE signaling axis, dehydroascorbic acid is oxidized and degraded to form 2,3-diketogulonic acid (2,3-DKG) rather than being regenerated into ascorbic acid (126), or it is directly absorbed into neurons via GLUT3, subsequently inducing neuronal apoptosis (127). Consequently, astrocytes have emerged as a promising therapeutic target, as their metabolic coupling with neurons not only protects neurons by removing excess FFAs (39) but also supplies antioxidant molecules to counteract oxidative stress.

While numerous studies have established that the antioxidant capacity of astrocytes is significantly superior to that of neurons, and that neuronal peroxidated lipids can be transported into astrocytes (128), several gaps in understanding persist. First, the mechanism by which neuronal peroxidated lipids are delivered to astrocytes remains unclear. Second, it is not fully understood whether astrocytic antioxidant molecules are transferred to neurons to combat oxidative stress, and if so, how this transfer occurs.

## 5 FAs and neuroinflammation in PD

Neuroinflammation is a common pathological feature of several neurodegenerative diseases and a key driver in PD pathogenesis (129). Preclinical studies indicate that neuroinflammation often precedes the onset of PD symptoms and is closely linked to dopaminergic neuron loss (130). Genetic evidence further suggests that PD-associated genes, such as *SNCA, LRRK2, DJ-1*, and *PINK1*, are involved in regulating neuroinflammatory responses (131). Autopsy studies in PD patients have revealed abundant activated microglia and elevated expression of pro-inflammatory mediators, including TNF-α, IL-1β, IL-6, and IL-1α (132). FAs play multiple roles in inflammation regulation and participate in central nervous system inflammatory responses through diverse molecular mechanisms. For instance, excessive exposure of the brain to very LCFAs can induce glial cells to convert them into excess sphingosine-1-phosphate, which in turn activates NF-κB signaling and promotes neuroinflammation (133). In contrast, the ω-3 PUFAs DHA and eicosapentaenoic acid have been shown to attenuate neuroinflammation and exert neuroprotective effects in mouse models by inhibiting NLRP3/ASC inflammasome signaling (93). Furthermore, DHA can reduce hippocampal neuroinflammation and oxidative stress by enhancing hippocampal insulin sensitivity, thereby improving cognitive dysfunction in aged rats fed a high-fat diet (88). Overall, these findings underscore the pivotal role of FAs in regulating neuroinflammation in PD and suggest that this pathway may be a crucial target in the broader context of neurodegenerative disease mechanisms. Elucidating the precise relationship between FAs and neuroinflammation in PD could provide a valuable theoretical foundation for developing novel therapeutic strategies against PD-related neuroinflammation.

Microglia, derived from the neural ectoderm, serve as the resident macrophages of the central nervous system (134). Under homeostatic conditions, their pro-inflammatory activity facilitates the clearance of abnormal substances and exerts neuroprotective effects (135). However, when microglia become chronically overactivated, they release excessive inflammatory mediators, and the persistent neuroinflammatory state contributes to PD pathogenesis. For example, M1-type neurotoxic microglia secrete IL-1α, TNFα, and C1q, which trigger the conversion of astrocytes into the A1-reactive phenotype. The soluble toxins released by A1 astrocytes subsequently damage neurons and accelerate PD progression (136). Emerging evidence indicates that microglial function is strongly modulated by FAs (**Figure 4**). SFAs generally promote a pro-inflammatory phenotype, whereas unsaturated FAs tend to confer anti-inflammatory effects (137). For instance, PA upregulates the expression of inflammatory mediators such as IL-1β, IL-6, TLR-4, and COX-2 in the microglial cell line HMC3, thereby exacerbating neurotoxicity. In primary mouse microglia, PA reduces levels of the anti-inflammatory cytokine IL-4, increases IL-1β expression, and induces the release of small extracellular vesicles that drive an immature dendritic spine phenotype in neurons, ultimately impairing cognitive function (138). Conversely, LA-treated microglia have been shown to counteract PA-induced neuroinflammation by modulating extracellular signal–regulated kinase phosphorylation and downregulating pro-inflammatory mediators, including IL-1β, IL-6, COX-2, and TLR-4 (139). FAs also influence neuroinflammation through the microbiota–gut–brain axis. Several studies have reported reduced short-chain FA levels in patients with PD (140). Short-chain FAs can modulate microglial activity via the gut–brain axis, promote the shift toward an anti-inflammatory M2 phenotype, and suppress the production of neurotoxic and pro-inflammatory factors (141). For example, Wang et al. demonstrated that DHA altered gut microbial composition, attenuated inflammation, and improved cognitive deficits in mice (95).

**Figure 4:**
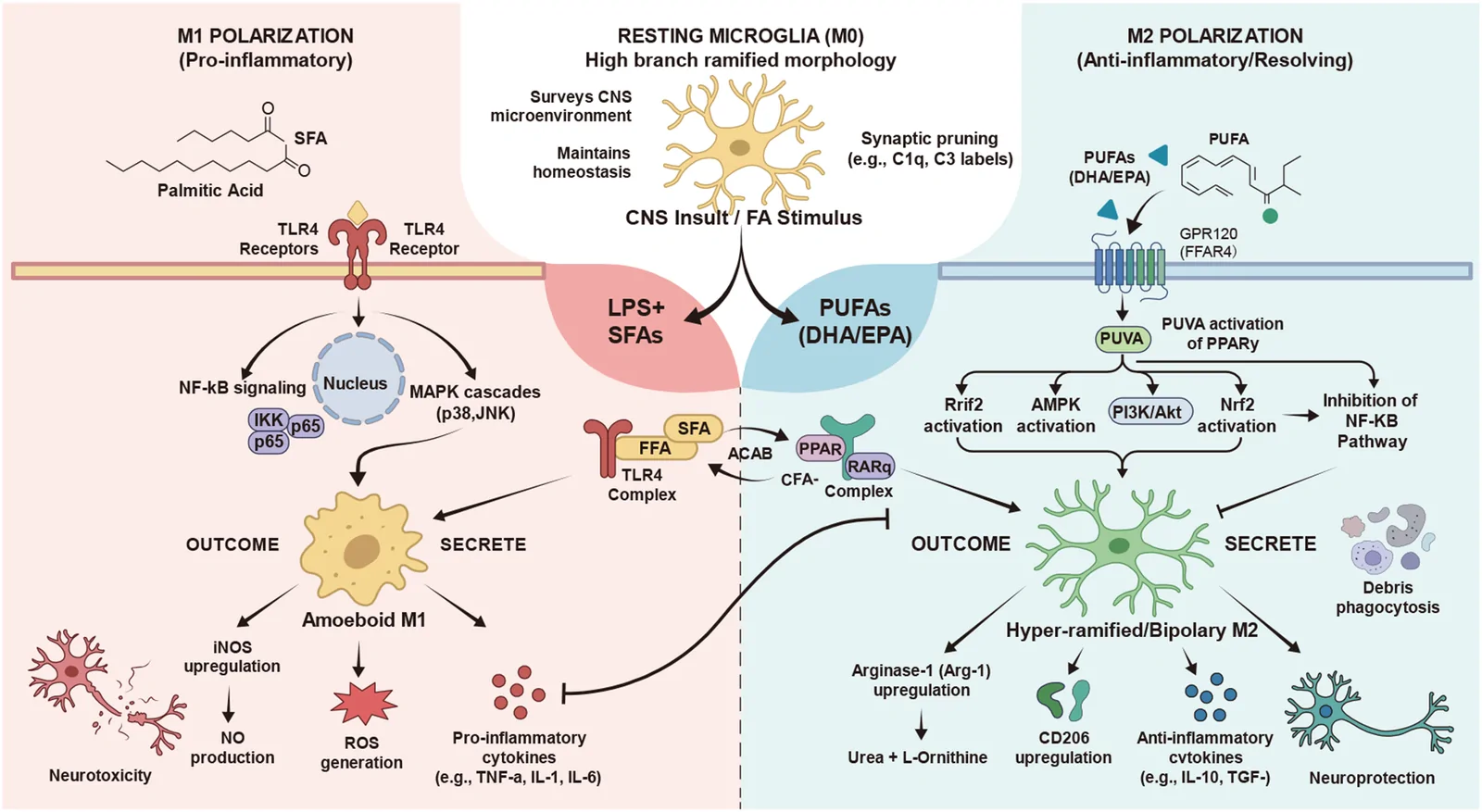
Fatty acid-dependent polarization of microglia in the central nervous system. Resting (M0) microglia maintain homeostasis and survey the CNS microenvironment. In response to saturated fatty acids (SFAs, e.g., palmitic acid) or LPS, microglia undergo pro-inflammatory (M1) polarization via TLR4-mediated activation of NF-κB and MAPK signaling. This drives amoeboid morphology, iNOS upregulation, ROS generation, and pro-inflammatory cytokine release (TNF-α, IL-1β, IL-6), leading to neurotoxicity. Conversely, polyunsaturated fatty acids (PUFAs, e.g., DHA/EPA) promote anti-inflammatory (M2) polarization through GPR120/PPARγ signaling. This activates pathways including AMPK, PI3K/Akt, and Nrf2, inhibits NF-κB, and induces hyper-ramified morphology, Arg-1/CD206 expression, anti-inflammatory cytokine release (IL-10, TGF-β), and enhanced debris phagocytosis, supporting neuroprotection. Created with Adobe Illustrator 2020.

Interestingly, a study indicated that the metabolic profile of microglia is closely linked to their inflammatory phenotype: M2 anti-inflammatory cells predominantly rely on FA oxidation and oxidative phosphorylation for energy production, whereas M1 pro-inflammatory cells shift toward glycolytic metabolism (142). Alterations in intracellular lipid metabolism in microglia play a role in regulating their phenotypic polarization and immune function (143). Targeted modulation of microglial substrate preference to promote oxidative phosphorylation and FA oxidation, thereby inducing M2 polarization, may help alleviate neuroinflammation (144). Moreover, the ω-6 PUFA/ω-3 PUFA ratio also influences microglial phenotypic transformation. In Fat-1 transgenic mice, a reduced ω-6 PUFA/ω-3 PUFA ratio promotes the production of TGF-β1 and COX-2 following LPS treatment, shifts microglia toward a more neuroprotective phenotype, and protects against LPS-induced pro-inflammatory cytokine production and cognitive deficits (145). Regulation of microglial lipid metabolism and metabolic reprogramming may represent a promising therapeutic target for neurodegenerative diseases. Since different FAs exert distinct effects on microglia, further research into their specific mechanisms of action has considerable potential. Modulating FA metabolism in the central nervous system to achieve anti-inflammatory effects may be an effective strategy for combating PD.

## 6 FAs and α-Syn in PD

α-syn is a soluble, small protein localized in synapses and perinuclear regions of the central nervous system, where it regulates neurotransmitter release, synaptic function, and synaptic plasticity (146). Under physiological conditions, α-syn primarily exists as soluble monomers, whereas membrane-associated physiological multimers, such as helical tetramers, demonstrate resistance to aggregation (147). However, under conditions of proteostatic stress or following post-translational modifications, misfolded α-syn monomers initiate nucleation-dependent amyloid aggregation. During this process, metastable soluble oligomers are formed in the lag-phase of primary nucleation. These heterogeneous oligomeric assemblies are regarded as the most neurotoxic entities in synucleinopathies, as they have the capacity to permeabilize cellular membranes, disrupt calcium homeostasis, and propagate pathology through prion-like seeding mechanisms. Oligomers may either dissociate back into monomers, follow an off-pathway to form non-fibrillar, dead-end aggregates, or continue along the on-pathway toward fibrillization (148). On-pathway oligomers further assemble into protofibrils, which are short, partially ordered fibril precursors that acquire a partial cross-β-sheet architecture with significant seeding potential. The subsequent elongation and lateral association of protofibrils result in the formation of insoluble, mature amyloid fibrils characterized by the canonical cross-β-sheet structure (149) (**Figure 5**).

In pathological tissues from patients with PD, numerous Lewy bodies containing abnormally aggregated α-syn fibrils as their main component have been observed (150). Aberrant accumulation and aggregation of α-syn oligomers or fibrils are a key driver of neuronal dysfunction and degeneration in PD (151). The pathogenic mechanisms by which α-syn aggregation contributes to PD are complex and involve mitochondrial dysfunction, oxidative stress, and neuroinflammation (150). In addition to aggregated α-syn, Lewy vesicles are enriched with lipid vesicles and membrane fragments (152). Increasing evidence suggests that both the physiological functions of α-syn and its pathological aggregation are closely linked to lipids. Structurally, α-syn comprises three domains: a positively charged N-terminal region (aa 1–60), a hydrophobic NAC domain (aa 61–95), and a negatively charged C-terminal domain (aa 96–140) (153). The N-terminal region, rich in basic residues, is highly homologous to the FA-binding region of FA-binding proteins and exhibits high affinity for negatively charged FAs (154). Under physiological conditions, α-syn binding to FAs regulates multiple processes in the central nervous system and helps maintain brain homeostasis. When α-syn binds to FAs on lipid membranes, it adopts a soluble α-helical secondary structure that regulates the equilibrium between membrane-bound and free α-syn, thereby maintaining its dynamic homeostasis (155). The continuous α-helical structure formed through this interaction is also critical for the assembly of soluble N-ethylmaleimide–sensitive factor attachment receptor complexes (156), which are essential for vesicular transport and membrane fusion, and contribute to the regulation of synaptic plasticity and neurotransmitter release (157).

However, interactions between α-syn and FAs under pathological conditions contribute to the pathogenesis of PD (**Figure 5**). The composition of FAs, including their content, saturation level, and carbon chain length, significantly influences the structural stability and oligomerization of α-syn (79). For example, an increase in unsaturated FAs such as DHA or oleic acid in the nervous system increased the expression of total α-syn and the formation of soluble α-syn oligomers and α-syn inclusion bodies in models of mutated or overexpression α-syn (80, 158). Mechanistically, DHA binding to α-syn induces its oligomerization through activation of the retinoic acid X receptor and PPAR-γ2 (80, 159). In contrast, TAG levels are significantly reduced in PD patients, and physiological TAG may help attenuate α-syn cytotoxicity by reducing abnormal accumulation of oleic acid and diacylglycerols (158). Notably, the toxic effects of DHA-induced α-syn appear contradictory to the neuroprotective effects of DHA reported in most studies. At present, there is no strong epidemiological evidence supporting a toxic role of DHA in α-syn-related PD pathogenesis, and further research is needed to clarify its interaction with α-syn. Interestingly, FA saturation exerts opposing effects on α-syn toxicity: saturated and monounsaturated FAs counteract the pro-aggregatory effects of PUFAs. Reducing FA unsaturation by inhibiting stearoyl-CoA desaturase has been shown to protect neurons from α-syn toxicity (160). Similarly, in a study on *Cryptomeria japonica*, silencing the Δ9 desaturase genes *fat-5* and *fat-7* improved α-syn expression and prevented neuronal damage (161). FA peroxidation products also contribute to α-syn oligomer formation (162). For instance, covalent binding of 4-hydroxynonenal to α-syn monomers induces toxic α-syn oligomers, reducing neuronal viability (163). In addition to directly modulating α-syn aggregation, the FA composition of cell membranes indirectly influences α-syn pathology by altering its membrane-binding affinity. The tendency of α-syn to aggregate is inversely proportional to its degree of membrane binding (164). At high lipid-to-α-syn ratios, α-syn binds to membrane surfaces without aggregating, whereas at low lipid-to-α-syn ratios, free monomeric α-syn undergoes rapid primary nucleation, accelerating aggregation (165). Moreover, α-syn and FAs regulate each other in a bidirectional manner. Overexpression of α-syn disrupts FA metabolism, leading to LD accumulation (166). In α-syn gene-ablated mouse astrocytes, a shift in FA distribution toward neutral lipids and an increase in neutral lipid mass have been observed (167). Additionally, α-syn mutants display reduced lipid-binding affinity, which is associated with increased propagation activity (168). Overall, the folding, aggregation, and distribution of α-syn are tightly linked to FA metabolism. The interactions between different FA types and various α-syn conformers under both physiological and pathological conditions remain incompletely understood. Dysregulated α-syn–FA interactions appear to be closely associated with pathological α-syn transformation and lipid metabolic alterations. Future studies should aim to define, in greater detail, the specific interactions between distinct FA species and α-syn variants.

**Figure 5:**
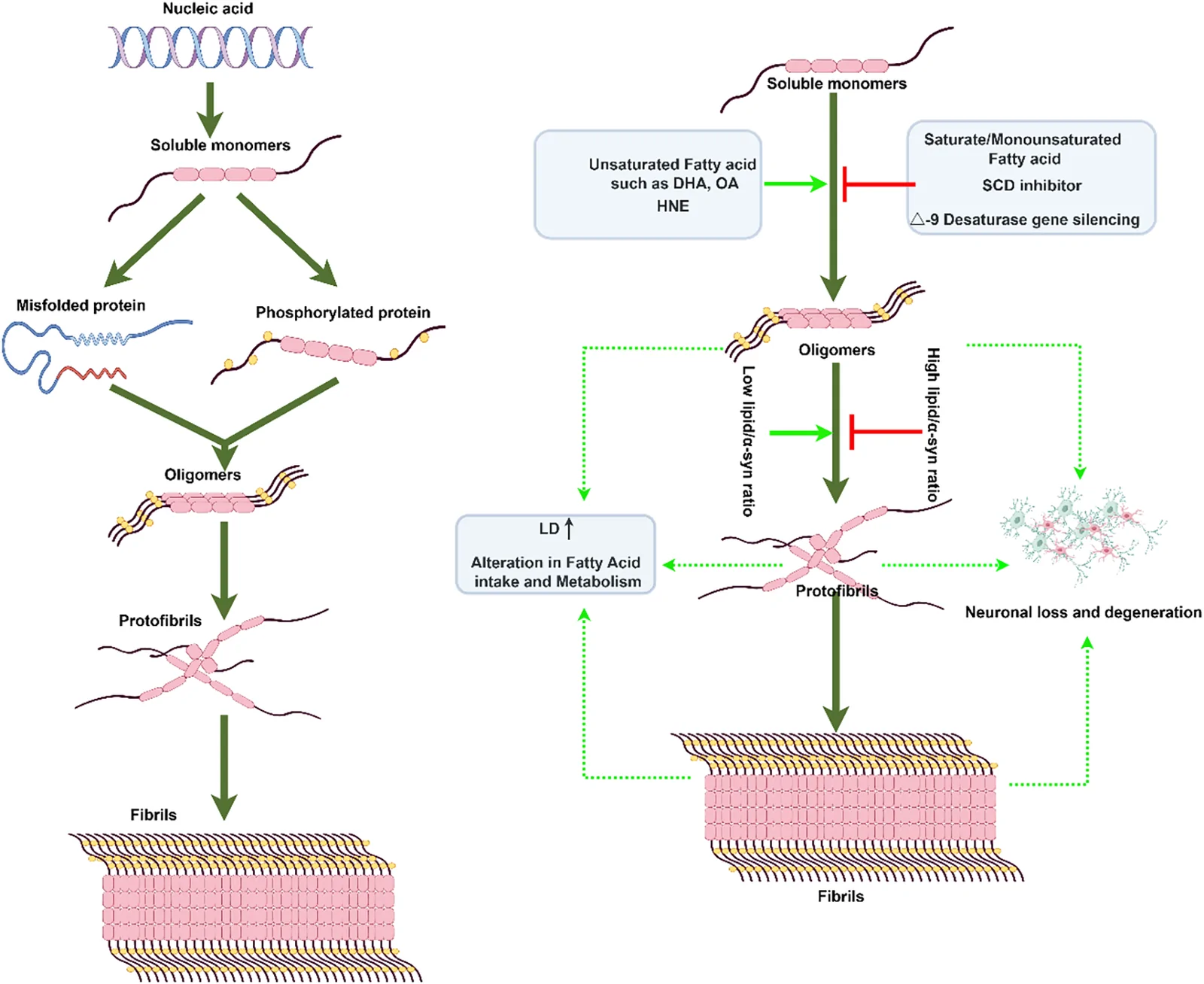
α-synuclein fibril formation and interaction between α-synuclein and fatty acids in the pathogenesis of PD. Under specific conditions, the natively unstructured monomer can convert into oligomers, which can further aggregate into protofibrils and finally into highly ordered cross-β-sheet fibrils (left panel). α-synuclein and fatty acids mutually interact and influence each other, contributing to disease onset and progression (right panel). Created with FigDraw 2.0 (Copyright Code: UTITR40aaf).

## 7 FA in modified proteins in PD

Post-translational lipidation constitutes a crucial mechanism for the regulation of protein function. Beyond their traditional roles in energy metabolism and membrane architecture, fatty acids are integral to the post-translational lipid modifications of proteins, particularly in the context of PD. This modification process entails the covalent attachment of fatty acids to specific amino acid residues, facilitating the association of proteins with cellular membranes and their involvement in a variety of biological processes. Notably, palmitoylation, which involves the reversible attachment of a 16-carbon saturated palmitic acid to cysteine residues via thioester bonds, has been identified as a pivotal regulatory mechanism in PD. This modification is mediated by palmitoyl acyltransferases (PATs) of the aspartate–histidine–histidine–cysteine (DHHC) family and can be reversed by acyl-protein thioesterases (APTs). The dynamic nature of palmitoylation permits the rapid modulation of protein function in response to cellular signals and environmental fluctuations. For instance, research has demonstrated that increasing cellular palmitoylation levels reduces α-syn accumulation and serine-129 phosphorylation, despite α-syn itself being incapable of palmitoylation due to the absence of cysteine residues. Mechanistically, pathogenic α-syn disrupts intracellular vesicle trafficking by excessively binding to vesicle membranes, thereby altering their curvature and interfering with their fusogenic properties (169–171). Concurrently, pathogenic α-syn accelerates the depalmitoylation of microtubule-associated protein 6 (MAP6), which facilitates vesicle transport by binding to vesicles in a palmitoylation-dependent manner (172). Myristoylation involves the irreversible attachment of a 14-carbon myristic acid to N-terminal glycine residues via amide bonds, a process catalyzed by N-myristoyltransferases (NMTs) (173, 174). This modification at the protein N-termini plays a critical role in cellular protein localization. For example, previous studies have found that the *src* gene product, p60src, of Rous sarcoma virus (RSV) is directly myristoylated and consequently targeted to the cell membrane (175). Consequently, researchers have devised a novel approach for addressing PD by augmenting the expression of neurotrophic factors or facilitating axonal regeneration within the adult nigrostriatal pathway. This strategy involves modulating the mTOR signaling pathway through the engineering of an N-terminal myristoylated variant of the survival signaling kinase Akt (176–178).

Despite many advances in elucidating protein lipidation, the intricate nature of these modifications and their regulatory mechanisms remain poorly understood. The advent of novel technologies, such as activity-based probes and mass spectrometry-based methodologies, has facilitated the systematic identification and quantification of lipidated proteins. These technological innovations have unveiled previously unrecognized lipidation events and offered valuable insights into the dynamic regulation of protein lipidation in neurodegenerative diseases such as PD.

## 8 Dietary FA and PD

There is increasing evidence that dietary habits constitute a significant modifiable risk factor for the development of PD. Higher SFA intake has been shown to induce chronic low-grade inflammation, oxidative stress, and blood–brain barrier dysfunction, all of which are associated with an elevated risk of PD (179). Yi et al. reported that high-fat diet mice exhibited reduced α-syn and brain-derived neurotrophic factor levels, increased tau46 and phosphorylated tau proteins, inhibition of the SIRT1/AMPK pathway, and disruption of autophagic flux, ultimately leading to cognitive decline (180). A high-fat diet also induces mitochondrial damage, promotes necrotic apoptosis, and causes neuronal loss, resulting in impaired motor function in mice (181). Excessive PA intake has been shown to disrupt non-essential amino acid synthesis pathways in Drosophila, leading to reduced glutamate synthase activity and increasing susceptibility to neurodegenerative diseases (182). The relationship between unsaturated FAs and PD appears to depend on the specific type consumed. Low dietary intake of ω-3 PUFAs is associated with reduced neuronal migration and neurogenesis, delayed cerebral glucose uptake and metabolism, and a heightened risk of PD (183–185). Modulating FA intake may partially alleviate both motor and non-motor PD symptoms. In a cross-sectional study, a diet rich in ω-3 PUFAs was associated with a reduced risk of mild cognitive impairment (186). The ratio of dietary ω-3 to ω-6 PUFAs also appears to be critical, with an appropriate increase in ω-3 PUFAs exerting neuroprotective effects and potentially delaying PD progression (187). Accordingly, nutritional or pharmacological interventions targeting ω-3 PUFAs or their derivatives may provide symptomatic benefits in PD. In recent years, the ketogenic and Mediterranean diets have garnered considerable attention for their potential impact on PD and its complications. The Mediterranean diet, characterized by high consumption of fresh vegetables and fruits, whole grains, legumes, seeds, and nuts, along with regular use of olive oil rich in high-quality FAs (28), has been linked to improved cognitive function in PD patients in a randomized controlled trial (188). The ketogenic diet, by reducing fat and glucose accumulation as energy reserves, promotes FA β-oxidation and ketone body production (189). Ketone bodies generated through this process have been shown to protect against mitochondrial dysfunction, attenuate neuroinflammation and oxidative stress, and shield neurons from free radical–induced damage (190). While the above studies suggest that dietary modulation presents a convenient and cost-effective strategy for preventing or decelerating the progression of PD symptoms, the conclusions are limited by a lack of precision. This imprecision arises from the susceptibility of observational studies to confounding factors such as race, geography, and lifestyle, among others. Therefore, further research is essential to elucidate the effects of total FA intake, as well as the specific contributions of different FA types, on the pathogenesis and progression of PD. Such research is necessary to more accurately define the potential benefits and risks associated with dietary interventions.

## 9 Is FA dysregulation a driver of PD?

The pathogenesis of PD entails sophisticated interactions between oxidative stress, neuroinflammation, mitochondrial dysfunction, disrupted cellular homeostasis, and metabolic imbalance. A number of existing mechanistic studies have confirmed that fatty acids are involved in all of the aforementioned processes (109). A critical unresolved question is whether FA imbalance serves as an initiator of pathological cascades (a driver) or occurs as a secondary effect of neuronal damage (a consequence). Epidemiological evidence suggests that FA disorders may play a role in the development of PD; however, the findings remain inconsistent. For instance, data from two studies—a study from the Swedish National March Cohort involving 41,597 participants and a prospective cohort study in Rotterdam with 5,289 participants—indicated that the intake of total fat, MUFAs, and PUFAs was significantly associated with a reduced risk of PD. Nevertheless, the results from the Swedish National March Cohort study demonstrated that higher consumption of saturated fats might be associated with an increased risk of PD (hazard ratio for the highest quartile vs. the lowest quartile: 1.41; 95% confidence interval: 1.04–1.90; p-value for trend: 0.03). In contrast, findings from the Rotterdam study suggested that dietary saturated fat, cholesterol, or trans-fat was not associated with the risk of PD (179, 191). Furthermore, a prospective cohort study and a Mendelian randomization analysis also suggested a causal inverse association of total cholesterol (TC), LDL-C, and triglycerides (TG) with PD risk (192). These investigations analyzed the relationship between fat and PD in diagnosed patients; however, they overlooked whether those PD patients had suffered from other pathological alterations induced by fat. Furthermore, the proportion of the population with incident PD was approximately 1% in these studies (435/41,597 and 51/5,289, respectively) (179, 191). We propose that these differences—including study duration, exposure environment, lifestyle, and dietary habits—may contribute to these inconsistent conclusions, and further basic studies in animals or humans are needed.

Meanwhile, compelling data from experimental and longitudinal clinical studies have also established lipid metabolism as a pivotal regulator that participates centrally in PD pathogenesis (19, 193–195) and may trigger its pathological cascades. For instance, Munoz-Pinto et al. provided evidence through fecal transplantation experiments that intestinal dysbiosis may precede the onset of PD by several years. Their study revealed that mice receiving fecal transplants from PD patients developed pathological phenotypes associated with PD (196). The researchers identified immune response disorders and disruptions of the intestinal barrier as the primary mechanisms underlying these observations. However, they did not explore the role of short-chain fatty acids (SCFAs), which are the main end products of bacterial fiber fermentation in the gut. This omission is significant, as substantial evidence from stool and plasma samples of clinical PD patients indicates that SCFA concentrations are altered in the early stages of PD (140, 193). Consequently, we propose that the dysbiosis-induced alteration in SCFA concentrations may contribute to the onset of PD. Additionally, Nuber et al. found that the levels of MUFA (C16:1, C18:1) and the ratio of α-syn between helically folded tetramers and natively unfolded monomers were disrupted in PD patient-derived glucocerebrosidase (GBA1) L444P mutant neurons and homozygous L444P mutant GBA1-knock-in (KI) mice; however, the motor deficit of GBA1-KI mice can be improved by decreasing the activity or content of stearoyl-CoA desaturase (SCD) using genetical tools or inhibitor (197). SCD is a critical regulator of lipogenesis that catalyzes the synthesis of monounsaturated fatty acids (MUFAs), mainly oleate (18:1n−9) and palmitoleate (16:1n−7) from saturated fatty acids, stearoyl-CoA (18:0) and palmitoyl-CoA (16:0), respectively. This finding showed that high levels of MUFA contributed to the formation of misfolded α-syn in mutant GBA1 PD patients and suggested that increased MUFA may be a driver of PD, although this study unrevealed the mechanism by which mutant GBA1 affects the activity or content of SCD. Furthermore, the disruption of fatty acid (FA) homeostasis through the inhibition of lipase (LIPE) has been shown to alleviate dopaminergic neurodegeneration in Caenorhabditis elegans models expressing human α-syn, thereby confirming that correcting FA imbalances can mitigate pathological outcomes (198). Genetic studies have also identified several fatty acid-specific risk loci, such as LIMP2, DGKQ, and ELOVL7, as being associated with PD (195). The expression of these genes influences cell membrane fluidity, membrane structure and integrity, and intracellular vesicle trafficking by regulating the synthesis, uptake, and specific types of fatty acids.

Collectively, the above findings indicate that FA dysregulation is not merely a consequence of neuronal damage but serves as an upstream trigger for α-syn aggregation, mitochondrial dysfunction, and neuroinflammation, which are core phenotypes of PD.

## 10 Discussion

As a central metabolic hub in the pathogenesis of PD, the FA metabolic network plays a pivotal role in driving disease progression by modulating key pathological processes, including neuroinflammation, protein misfolding, and oxidative stress. Precise regulation of FA metabolic fluxes can maintain a dynamic equilibrium between toxic and protective lipid species, offering a potential therapeutic avenue that may overcome the limitations of current treatments, which often have suboptimal efficacy and notable side effects. However, in contrast to advances in proteomics and genomics, lipidomics still faces significant technical challenges. The structural heterogeneity of lipid molecules, combined with their dynamic modifications, complicates the elucidation of their specific roles in PD. Future research that systematically maps the dynamic remodeling of FA metabolic networks across distinct pathological stages of neurodegenerative diseases could yield critical insights. Such analyses may enable targeted attenuation or clearance of α-syn aggregates, reduction of astrocyte- and microglia-mediated inflammation, and mitigation of oxidative damage leading to dopaminergic neuronal loss. This approach holds promise for developing novel therapeutic strategies that modulate the metabolic microenvironment to influence PD pathology. Moreover, we propose that the following research directions outlined herein could hold substantial significance for the clinical application of FA regulation: (1) undertaking stage-specific lipidomic profiling to elucidate dynamic lipid alterations across various stages of PD; (2) engaging in mechanistic studies to validate causative relationships within fatty acid-related pathways, moving beyond mere correlative observations; and (3) progressing preclinical and translational research focused on targeting neuroinflammation for the development of PD therapeutics.

## Glossary

PD: Parkinson’s disease
ROS: reactive oxygen radicals
FAs: fatty acids
LDs: lipid droplets
ω-3 PUFA: omega-3 polyunsaturated fatty acids
ω-6 PUFA: omega-6 polyunsaturated fatty acids
PUFAs: polyunsaturated fatty acids
DHA: docosahexaenoic acid
α-Syn: alpha-synuclein
MPTP: 1-methyl-4-phenyl-1,2,3,6-tetrahydropyridine
MC1R: melanocortin 1 receptor
Nrf2: nuclear factor erythroid 2-related factor 2
PP2A: protein phosphatase 2A
LCFA: long-chain fatty acids
LRRK2: leucine-rich repeat serine/threonine-protein kinase 2
FABPs: fatty acid binding proteins
FAT/CD36: fatty acid translocator
TAGs: triacylglycerol
SFAs: saturated fatty acids
PA: palmitic acid
ACC: acetyl-CoA carboxylase
FFAs: free fatty acids
ApoE: apolipoprotein E
DGAT-2: diacylglycerolacyltransferase-2
PRKN/Parkin: parkin RBR E3 ubiquitin protein ligase
RELA: RELA proto-oncogene, NF-κB subunit
HDAC8: histone deacetylase 8
6-OHDA: 6-hydroxydopamine hydrochloride
GLUT3: glucose transporter 3
NLRP3: NOD-like receptor thermal protein domain associated protein 3
ASC: apoptosis-associated speck-like protein containing a CARD
IL-1α: interleukin-1α
TNFα: tumor necrosis factor α
C1q: complement C1q
TLR: Toll-like receptor
COX-2: cyclooxygenase-2
PPAR-γ2: peroxisome proliferator-activated receptor gamma 2
PATs: palmitoyl acyltransferases
DHHC: aspartate–histidine–histidine–cysteine
APTs: acyl-protein thioesterases
MAP6: microtubule-associated protein 6
NMTs: N-myristoyltransferases
SCD: stearoyl-CoA desaturase.
